# Ischemic stroke in Peru: a scoping review of trends, outcomes, and evidence gaps

**DOI:** 10.3389/fepid.2026.1926861

**Published:** 2026-08-28

**Authors:** Christoper A. Alarcon-Ruiz, Jesus Gutierrez-Arratia, Lessly Concha-Querevalú, Erick Barrientos-Ventura, Jacqueline Moreno-Arias, Ricardo Otiniano-Sifuentes, Rosa Ecos, Joseph R. Zunt, J. Jaime Miranda

**Affiliations:** 1 Instituto Nacional de Ciencias Neurológicas, Lima, Peru; 2 Unidad de Investigación Para la Generación y Síntesis de Evidencias en Salud, Universidad San Ignacio de Loyola, Lima, Peru; 3 Facultad de Ciencias de la Salud, Universidad San Ignacio de Loyola, Lima, Peru; 4 Departamento de Enfermedades Neurovasculares y Metabólicas, Instituto Nacional de Ciencias Neurológicas, Lima, Peru; 5 Departments of Neurology, Global Health, Medicine (Infectious Diseases), and Epidemiology, University of Washington, Seattle, WA, United States; 6 Sydney School of Public Health, Faculty of Medicine and Health, The University of Sydney, Sydney, NSW, Australia; 7 CRONICAS Centre of Excellence in Chronic Diseases, Universidad Peruana Cayetano Heredia, Lima, Peru

**Keywords:** epidemiology, ischemic stroke, outcome, prevention, public policy

## Abstract

**Background:**

Peru faces a growing mismatch between the increasing burden of ischemic stroke and the health system's capacity to respond.

**Aim:**

We aimed to systematically map the primary research literature on ischemic stroke in Peru using a socio-ecological framework, characterize the current evidence base, quantify key epidemiological outcomes, and identify critical knowledge gaps to inform a national research agenda.

**Methods:**

We conducted a scoping review using the SPIDER framework and searched four databases through April 2025. We included original primary studies of Peruvian participants of any age with ischemic stroke, including observational, interventional, qualitative designs, and case reports. Study selection and data extraction were performed in duplicate. Findings were narratively synthesized across socio-ecological levels (individual, interpersonal, organizational, community, and policy/environmental). Key epidemiological outcomes were pooled using random-effects meta-analyses.

**Results:**

Of 1210 records, 119 studies were included (131,424 participants), with 75% conducted in Lima. Ischemic stroke represented 62% of all stroke cases; 44% were women. Overall mortality was 12%. Large-vessel atherosclerosis (27%) and cardioembolic (24%) were common etiologies, but undetermined causes remained the highest (30%). The pooled thrombolysis rate was 2.9%, with no reported access to mechanical thrombectomy in observational studies. Qualitative studies identified barriers to timely care, poor guideline adherence, caregiver burden, and centralization of services. Quality-improvement efforts showed limited impact. Community prevention and digital innovations were tested in small pilots without scale-up. Policy-level evaluation was scarce.

**Conclusion:**

Research regarding stroke in Peru has increased but remains geographically centralized and structurally misaligned with the national burden. Major gaps persist in diagnostic capacity, implementation research, rehabilitation, caregiver support, and policy evaluation. A nationally coordinated and regional distributed research and system-level strengthening of the stroke program are urgently needed.

**Systematic Review Registration:**

(https://doi.org/10.17605/OSF.IO/7WYX8).

## Introduction

Stroke remains among the leading causes of death and long-term disability worldwide, with associated burden rising substantially over the past three decades (1, 2). In 2021, an estimated 11.9 million incident strokes occurred worldwide, 65% of which were ischemic stroke (1). Recent population-based estimates report an age-standardized incidence of ischemic stroke in Peru of approximately 1.65 per 10,000 persons yearly and a marked altitudinal gradient, highlighting Peru's unique geographic and demographic determinants of risk and care access (3). Ischemic stroke has different pathophysiological mechanisms, acute management protocols, and secondary prevention strategies, that often differ significantly from those of hemorrhagic stroke (4).

The burden of ischemic stroke is disproportionately concentrated in low- and middle-income countries, which account for approximately 86% of ischemic stroke deaths and 87% of disability-adjusted life years (5). In Latin America and the Caribbean, rapid demographic and epidemiological transitions, layered onto pre-existing health-system limitations, continue to maintain high morbidity of ischemic stroke (6). These limitations including restricted thrombectomy capacity, nascent telemedicine networks, and limited access to timely rehabilitation, highlight the need for context-specific implementation research (7, 8).

Peru exemplifies these challenges in a unique epidemiological setting. Local data show a critical gap in ischemic stroke care: at the national tertiary referral neurology institute in Lima, less than 2% of patients with ischemic stroke received intravenous alteplase between 2009 and 2016, a pattern driven largely by delayed presentation and system barriers (9). Physical and human health resources are highly centralized in urban settings and care pathways are fragmented (10, 11). Access to advanced imaging and mechanical thrombectomy is limited outside the capital and largely concentrated in tertiary centers, and rehabilitation services are underdeveloped (11, 12).

Unlike global or regional analyses, country-level evidence mapping in a lower-middle-income setting allows identification of where the research data is structurally misaligned with the actual disease burden and geographic distribution of care (13). The mismatch between a growing disease incidence and burden (3) and the country's limited capacity to respond (12, 14) demonstrates the urgency of a targeted research agenda in Peru. This scoping review has a single primary objective: to systematically map the existing primary literature on ischemic stroke in Peru using a socio-ecological framework, in order to characterize the evidence base, quantify key epidemiological outcomes where feasible, and identify the most critical knowledge gaps that should guide a national research agenda. This framework was selected because we aimed to assess the determinants of ischemic stroke prevention and care extend beyond the individual clinical level to interpersonal, organizational, community, and policy/environmental factors.

## Methods

### Study design and protocol

This study reports a scoping review in accordance with the PRISMA checklist for Scoping Reviews (Supplementary Table S1). The protocol was previously developed by the authors and is available on OSF.io (https://doi.org/10.17605/OSF.IO/7WYX8).

We followed a mixed quantitative and qualitative approach utilizing the SPIDER framework (15) to guide the scoping and synthesis of evidence. Within this framework, our Sample of interest was the Peruvian population; the Phenomenon of Interest was specifically the ischemic stroke; the eligible Design included published literature of any research design; and the Research Type encompassed primary quantitative or qualitative research data, including case reports. The resulting body of evidence was subjected to Evaluation following a socio-ecological approach, which structured the synthesis around five key categories for determinants: 1) Individual, 2) Interpersonal, 3) Organizational, 4) Community, and 5) Policy and environment (16). Unlike other frameworks focused primarily on intervention planning, such as PRECEDE-PROCEED (17), or on sequential clinical care pathways (18), the socio-ecological approach allows heterogeneous evidence to be mapped across multiple levels of the health system, consistent with the objective of identifying structural gaps in Peru.

### Search strategy and selection criteria

We systematically searched in Scopus, Web of Science Core Collection, Scielo via Web of Science, and PubMed/Medline databases, using the terms of “Stroke”, “Ischemic cerebrovascular event”, “Acute ischemic attack”, “Apoplexy”, “Infarct”, and “Peru”, to ensure maximum initial comprehensiveness. Detailed search strategies for each database can be found in Supplementary Table S2. The search date was carried out on January 17th, 2024, and was updated only in Scopus database on April 4th, 2025, due to methodological efficiency, avoiding duplicated efforts, and its superior coverage for retrieving the highest number of results, usually overlapped, compared with PubMed/Medline and Web of Science.

We included original studies conducted in human participants of any age diagnosed with ischemic stroke or reports ischemic stroke as an outcome. Eligible study designs included observational studies, interventional studies, and qualitative research. The latter involved patients, caregivers, or healthcare professionals engaged in the care of individuals with any type of stroke. Also, we included studies that proposed novel rehabilitation interventions for patients with stroke, ischemic or hemorrhagic, because their disability is similar. Only studies that reported specific information on persons within the Peruvian territory were included. Availability or reporting of neuroimaging was not a prespecified eligibility criterion.

Consistently with a scoping review framework that explicitly values breadth of coverage, case reports and case series may present uncommon or complex stroke etiologies that would otherwise be invisible in aggregate epidemiological datasets. While conference abstracts published in peer-reviewed scientific journals sometimes represent the only primary publications available for describing new technological interventions or small pilot interventional studies. Thus, we included case reports and case series that describe uncommon etiologies or treatments of ischemic stroke, as well as conference abstracts published in peer-reviewed scientific journals for novel rehabilitation studies and pilot interventional studies.

In case of studies that included both ischemic and hemorrhagic stroke, those were included if they presented separate data for only ischemic stroke. The following types of studies were excluded from the review: studies focused on global ischemic cerebrovascular disease (e.g., post-anoxic encephalopathy or global cerebral ischemia), editorials, letters to the editor without original results, and reviews that did not report primary data. Studies focused on cerebral venous thrombosis or venous infarction were also excluded because these conditions have distinct pathophysiological mechanisms, diagnostic pathways, and acute and secondary treatment strategies compared with arterial ischemic stroke. The review therefore focused specifically on arterial ischemic stroke.

### Study selection and data extraction

Initially, the results from each search were exported to the free online Rayyan QCRI software (https://rayyan.qcri.org; Qatar Computing Research Institute, Qatar Foundation, Qatar) and duplicated references were eliminated. Then, two independent researchers evaluated the titles and abstracts and then conducted a full-text review as a secondary step (CAA-R and JG-A). The discrepancies were resolved by consensus in both processes.

Data from the included studies were independently extracted by the six researchers paired in three independent groups (CAA-R and JG-A, RO-S and JM-A, RE and LC-Q) using a data collection form in Microsoft Excel (Microsoft, Washington, USA) designed for each study design. For case reports, we collected demographic (sex and age) and clinical data [symptoms, vascular territory, TOAST etiology reported by the article and assessed by the reviewer [large artery atherosclerosis, cardioembolic, small vessel, other determined, or undetermined] (19), treatment, and follow-up]. For observational studies, we collected population characteristics (location, time period, number of included subjects, and number of subjects for each etiology following the TOAST classification and as reported by individual studies, when available), magnetic resonance imaging (MRI) or advance perfusion imaging use, epidemiological outcomes (prevalence, incidence, mortality, disability), and clinical characteristics (arrival time to hospital, etiology, reperfusion rates, COVID-19 related information, associated factors or outcomes). We did not find studies reporting a different classification system for ischemic stroke rather than TOAST criteria.

For qualitative studies, we collected study population and different subjects' reports (healthcare access, rehabilitation, psychological impact, costs, personal experiences, and COVID-10 related information). Finally, for intervention studies we collected population information, type and details for intervention, and main results. All data collected were retrieved by two independent investigators (CAA-R and JG-A, RO-S and JM-A, RE and LC-Q), and in case of discrepancies, they were solved by consensus.

### Synthesis of results

Most findings were summarized narratively following a socio-ecological health approach, dividing the results into five levels: Individual, Interpersonal, Organizational, Community, and Policy and environment. Studies were classified according to the primary level at which the determinant or barrier operated; when findings overlapped across levels, classification was resolved through reviewer discussion and consensus. We also produced descriptive frequency analyses of study design, year of publication, and geographic setting, using a curated dataset derived from the extraction table. For quantitative synthesis we prespecified several outcomes: (i) proportion of ischemic stroke among all cerebrovascular events, (ii) proportion of ischemic stroke within cohorts recruited for another disease, (iii) proportion of women, (iv) mortality rate, (v) thrombolysis rate, and (vi) etiology classification among ischemic stroke cases. We pooled outcomes when at least three studies reported compatible numerators and denominators from the same source population after cleaning and harmonization. When multiple reports referred to the same cohort, we retained the record with the largest valid denominator or the most complete information and removed obvious duplicates. If a report provided several time periods, we used study year pairs only when period-specific denominators were explicit; otherwise, a single overall estimate was retained. Studies with incompatible definitions or unresolved inconsistencies were excluded from that synthesis but retained for the narrative description. All quantitative syntheses were implemented in R using the *meta* and *metafor* packages, with modeling details provided in the Statistical analysis section.

### Prespecified outcomes

We synthesized several key outcomes related to ischemic stroke in Peru. Primary metrics included: i) proportion of ischemic stroke among all cerebrovascular events, calculated using the sum of ischemic and hemorrhagic strokes within the same source population as the denominator. To capture ischemic stroke burden as a complicating condition in non-stroke-primary study populations, we evaluated ii) proportion of ischemic stroke within non-stroke cohorts recruited for other diseases or conditions, using the total size of the non-stroke cohort as the denominator. Studies that only enrolled stroke patients were excluded from this specific synthesis.

To characterize the patient population and acute management, we also calculated iii) proportion of women among all ischemic stroke cases, and iv) proportion of deaths (both intrahospital or after discharge deaths) among all ischemic stroke cases. Regarding acute care access, we quantified v) proportion of thrombolysis utilization among all ischemic stroke cases. Finally, we aimed to map the underlying causes by determining vi) proportion of each stroke etiology (large artery atherosclerosis, cardioembolic, small vessel, other determined, or undetermined) among the total number of ischemic stroke cases.

### Statistical analysis

Proportions were modeled with *metaprop* on the logit scale (PLOGIT) and were backtransformed for presentation. Random-effects models were the primary approach. Between-study variance (*τ*^2^) was estimated with restricted maximum likelihood, and confidence intervals used the Hartung-Knapp adjustment. To enable computation of overall estimated proportion, in studies with zero-event cells, a continuity correction of 0.5 was applied uniformly, when required. We reported *I*^2^, *τ*^2^, and a 95 percent confidence interval for each proportion metanalysis. Common-effect estimates were provided as a reference.

### Frequency analysis and graphics

A consolidated dataset captured publication year, study design, geographic distribution, and center type. Annual counts by design were computed and plotted as stacked bars to show temporal trends. Geographic distribution and the share of single-center and multicenter studies were summarized from the same curated dataset to maintain internal consistency across the study characteristics table and figures.

### Software and reproducibility

All quantitative syntheses were implemented in R using the meta and metafor packages. Data handling and figure generation used tidyverse utilities as well as readxl, janitor, stringr, stringi, and openxlsx for spreadsheet input and output. Forest plots reflect the logit transformation, restricted maximum likelihood for *τ*^2^, the Hartung-Knapp adjustment, and 95 percent confidence intervals. Curated included and excluded datasets, pooled summaries, influence tables, and figures were exported programmatically and are available upon request together with the scripts and output directories.

## Results

### Study selection and characteristics

**Figure 1:**
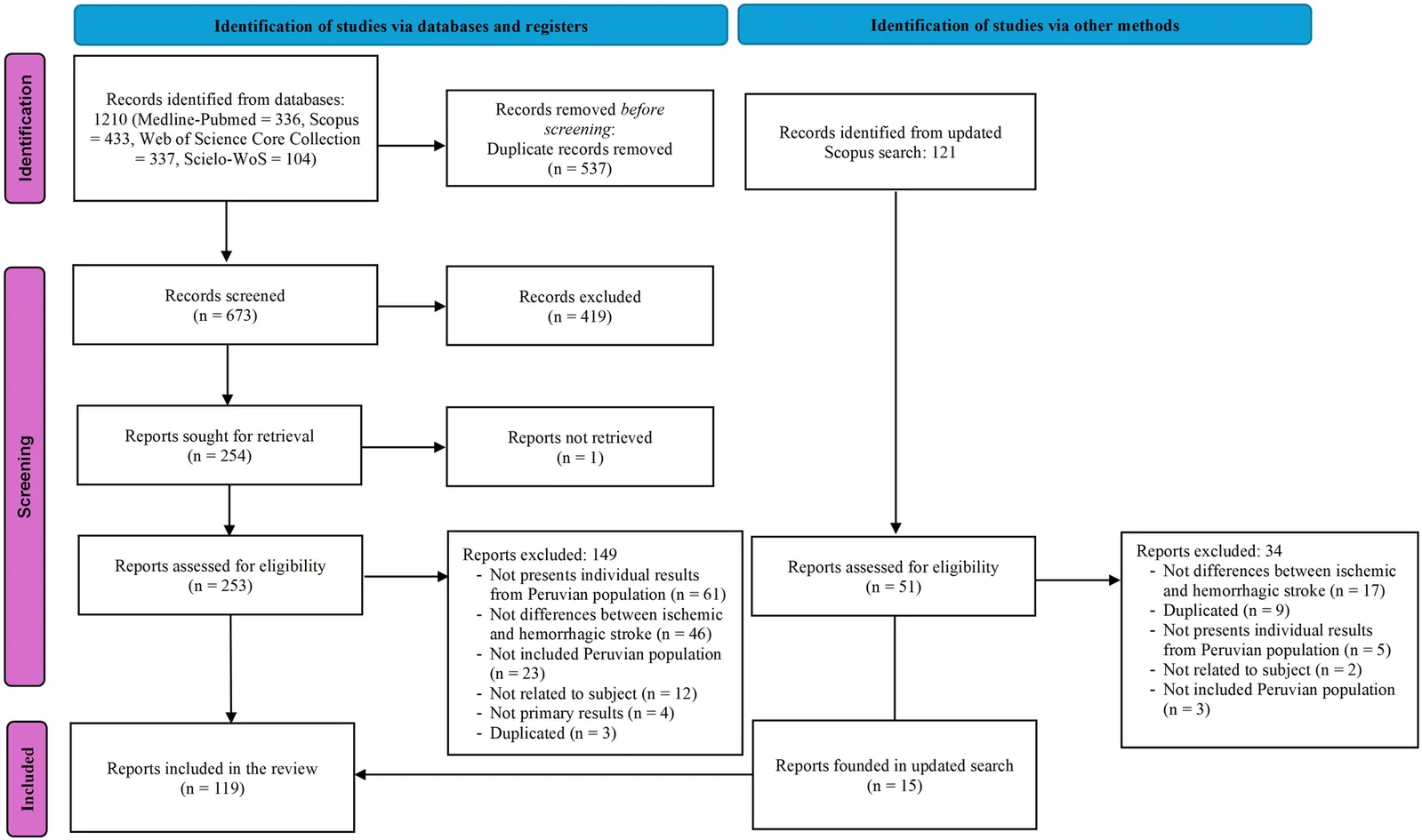
PRISMA flowchart for study selection.

**Figure 2:**
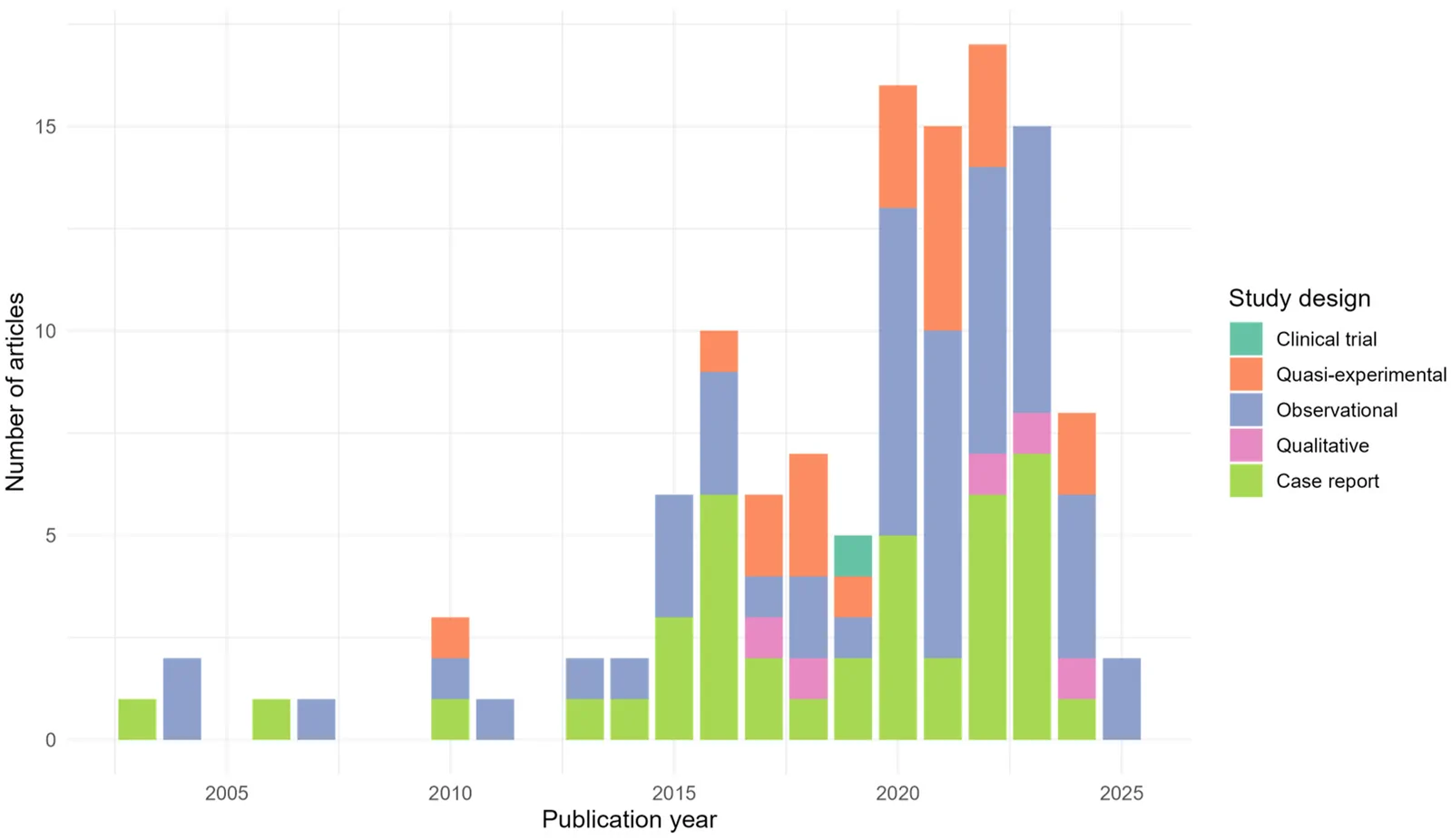
Study distribution according to study type.

We identified 1,210 records through database search, and after removing duplicates, 673 records were screened. Two hundred fifty-three records were evaluated in full text, and 149 records were excluded. We identified five randomized clinical trials for ischemic stroke prevention, two for acute treatment, one for secondary prevention and one for rehabilitation, but all were excluded because they did not present independent results of the Peruvian population (reasons provided in Supplementary Table S3). Following an updated search in Scopus, 119 articles were included in this scoping review (Figure 1). The final sample comprised 53 observational studies (*n* = 131 370 study participants), 38 case reports documents (*n* = 54 subjects), 21 quasi-experimental studies, five qualitative studies, and one cluster randomized clinical trial. Among all, 75.4% were from Lima. Most of these studies were published during or after 2020 (Figure 2).

### Individual level: case reports

**Figure 3:**
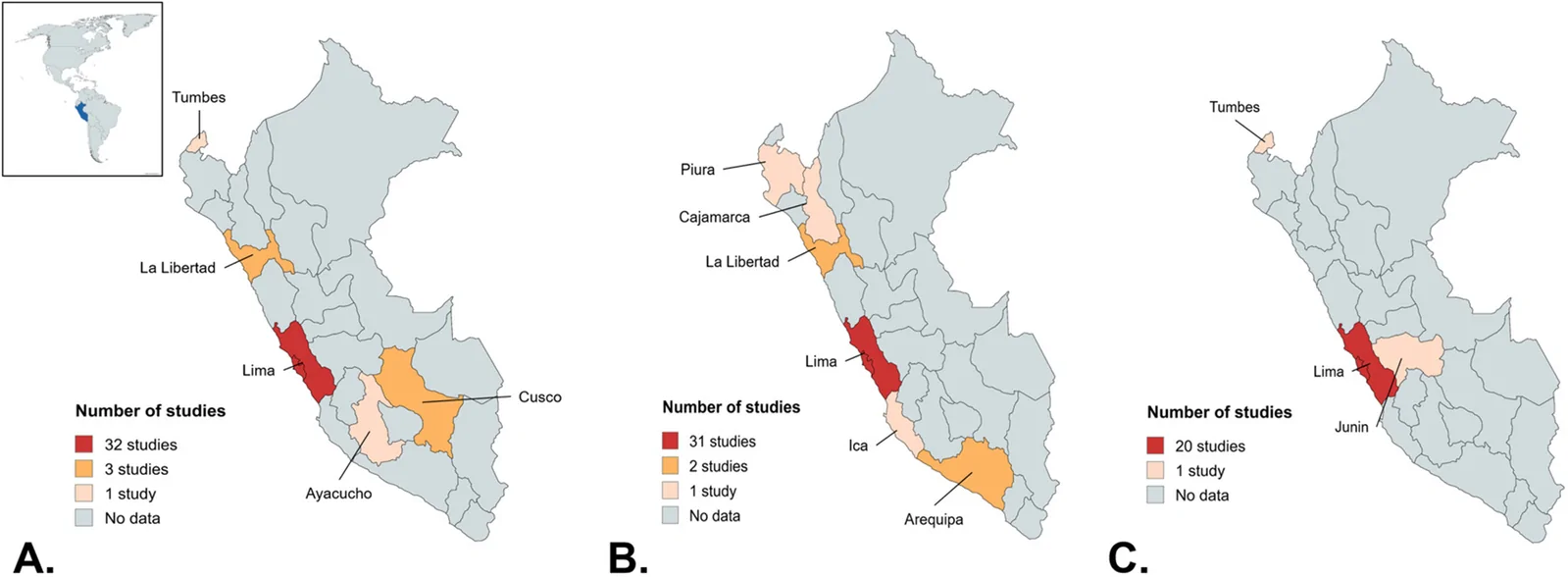
Frequency of studies according to region by study type. **(A)** Observational studies; **(B)** Case reports; **(C)** Quasi-experimental studies.

The included cases represent a substantial portion of the available primary literature on ischemic stroke in Peru, particularly for uncommon etiologies. Reports were retrospectively collected and were published between 2003 and 2024. Among the 54 reported individuals, the median age was 45 years old with range from 5 to 91 years old, with females as 53%. Cases were reported mostly from institutions in Lima (87%), while two cases were from Arequipa and two from La Libertad, and one case from Cajamarca, Ica and Piura, each (Figure 3B). Most of the cases (80%) were ischemic strokes of anterior circulation. Undetermined etiology was the most frequent diagnosis (48%), followed by other determined etiology (35%) and cardioembolic etiology (6%). However, there were 7 cases where the etiology mentioned by the report were cardioembolic or other determined etiology, but after the critical revision, we could not find enough evidence to confirm those etiologies. The most common symptoms were motor deficit (83%), cognitive or behavioral impairment (35%), headache (15%), and epileptic seizures (13%).

### Individual level: epidemiological studies

The compiled database of 53 observational studies of ischemic stroke in Peru demonstrated substantial heterogeneity across populations and reported outcomes, limiting direct comparability but offering a broad portrait of the national research landscape. The aims of these studies were as follows.

a) studies where ischemic stroke was the primary recruitment criterion (34%); (b) studies where ischemic and hemorrhagic stroke patients were included (20%); and (c) studies where ischemic stroke was a secondary or incidental outcome within a non-stroke cohort (22%) or in a general population (24%). Among this last general population group, most studies used nationwide databases.

Among 53 observational studies, most were from a single center (79%), conducted in Lima (76%), and recruited participants between 2012 and 2022 (Table 1). The representation of rural, high-altitude Andean or Amazonian settings was almost null (Figure 3A) (20–22). Many of these studies reported different epidemiological estimates (frequency and mortality), but methods, sample size, denominators, and study periods varied widely. The pooled proportion of ischemic stroke was 65% among all ischemic and hemorrhagic stroke cases (*n* = 36,513) (Supplementary Figure S1), however, there was not specific data regarding sex and age among most of these studies. Across studies with available data, women represented 44% of ischemic stroke cases (*n* = 5,821) (Supplementary Figure S2); mean age among all ischemic stroke cases (*n* = 3,396) was 66·9 years old (Supplementary Figure S3); and 8% of patients with other non-stroke conditions like COVID-19, cardiovascular diseases, and systemic infections (*n* = 104,693) experienced an ischemic stroke (Supplementary Figure S4). Regarding etiology, large-vessel atherosclerosis was the most frequent determined etiology (25%), followed by cardioembolic (23%), small-vessel disease (23%), and other determined etiologies (3%). However, the pooled proportion of undetermined etiology was the highest at 30% (Supplementary Figures S5–S9). Only two observational studies from Lima reported the number of patients with MRI use (23, 24), and no study reported the use of advanced perfusion imaging.

**Table 1:** Observational studies characteristics regarding ischemic stroke in Peru.

Variable	Categories	Total	General population	Ischemic or hemorrhagic stroke	Only ischemic stroke	Non-stroke cohorts (Other pathology)
Centers	One center	33	1	8	13	11
	Multicentric	9	2	1	5	1
	Nation-wide secondary data	11	8	2	0	1
Region	Lima	32	1	7	14	10
	Cusco	3	0	0	1	2
	La Libertad	3	0	1	2	0
	Tumbes	1	1	0	0	0
	Ayacucho	1	0	1	0	0
Study period	Before 2012 (Pre-thrombolysis)	17	2	5	5	5
	2012	8	0	1	3	4
	2013	7	0	1	3	3
	2014	7	0	1	3	3
	2015	12	3	1	5	3
	2016	14	3	3	5	3
	2017	11	2	3	2	4
	2018	9	1	2	3	3
	2019	10	2	2	3	3
	2020	12	1	2	4	5
	2021	10	2	1	3	4
	2022	5	1	0	2	2

Among patients with ischemic stroke, overall mortality was 12% (Supplementary Figure S10), of which most occurred while hospitalized. Only one study reported one-year mortality (25). There was no information regarding mortality stratified by imaging modality, infarct volume, or imaging-defined stroke severity. Disability and other clinical outcomes were reported inconsistently across studies, but several series highlighted a high burden of post-stroke disability (9, 22, 26–31) Risk-factor data emphasized hypertension, dyslipidemia, cardiovascular disease, hormonal contraceptive, and aging as recurrent contributors for ischemic stroke development (32, 33). Additionally, there were reports of some risk factors for worse outcomes among ischemic stroke patients, such as inflammatory biomarkers (high C-reactive protein, high with blood count, and low acid uric levels) (30, 34, 35) and intrahospital complications (hydrocephalus, hemorrhagic conversion, infections) (33, 34, 36). Compared to hemorrhagic strokes, patients with ischemic stroke had lower mortality and shorter hospital stays^(^29, 37, 38). Finally, a few studies reported the association between ischemic stroke with depression (39) and cognitive impairment or dementia (40, 41).

### Individual level: feasibility of computer-based innovations

Eighteen studies proposed innovative and accessible robotic or computer-assisted solutions for post-stroke patients, mostly from Lima (Figure 3C). These included eight reports of brain-computer interphase validated in post-stroke patients to be used in smart-homes or other adaptive software for their lifestyle activities (42–49) Eight additional studies reported initial validation of novel interventions for upper limb rehabilitation using exoskeletons (50–54), virtual reality (55, 56), and an orthosis (57). While other studies described the telerehabilitation communication system for post-stroke patients (58), and a novel algorithm for gait assessment in post-stroke patients with tactile impairment in lower legs (59). Most of them were software presentations with small sample size of patients with stroke and healthy controls to pilot them. Despite these projects representing a promising advancement in healthcare innovations, they remained at a very early stage of development with no assessment within a large-scale population.

### Interpersonal level: caregivers and healthcare providers

Five qualitative studies reported the challenges of caregivers and healthcare providers caring for post-stroke patients, all from Lima. Caregivers' worries and fears were linked to their patients' new disabilities and the presence of invasive instruments such as nasogastric tube (60). Additionally, caregivers faced reduced social activity and unexpected financial burdens (61) including hiring private services to overcome care gaps (62). This new situation causes increase caregiver burden (62) and emotional stress and depressive symptoms, which increase their needs to see a mental-health professional (61).

Most healthcare providers lack correct training for stroke rehabilitation (63). Caregivers reported receiving only oral, not written, information about rehabilitation with no practical training in post-stroke care tasks after discharge (64). While healthcare providers indicated that only nurses were responsible for delivering this information (63). However, interventions with interprofessional education may increase the knowledge and confidence of healthcare providers and caregivers for care of post-stroke patients (64).

### Organizational level: access to healthcare in stroke

Patients described multiple barriers to timely emergency care, including poor awareness of stroke (64) and limited understanding of how the health system works (64). Five observational studies reported the time from symptom onset to hospital arrival in patients with ischemic stroke: In patients who received thrombolysis, mean time was 134.2 min (9, 36), while in other studies, arrival times ranged between 3 h to 7 days (35, 65, 66). Among ten studies reporting thrombolysis rates, overall frequency was 2.9% (Supplementary Figure S11). No study reported data regarding indications for or access to mechanical thrombectomy, although the procedure is performed in few tertiary public and private centers in Peru (67, 68).

During hospitalization, caregivers highlighted communication challenges with healthcare providers, such as nonfunctioning phone lines, limited information sharing, inconsistent explanations between providers, and at times impolite interactions (63). In addition, after hospital discharge, caregivers reported feeling underprepared to manage care of the patients and noted geographic disparities in access to dysphagia services (60), and barriers to obtaining a referral for specialized stroke management using the formal referral pathway (63).

### Organizational level: adherence to recommendations

One local study reported mean adherence to AHA/ASA stroke performance measures of 47%. Only five of 15 AHA/ASA stroke performance measures were achieved at a level of 85% or higher: antithrombotic treatment at discharge, antithrombotic treatment within 2 days of hospitalization, discharge on statins, rehabilitation assessment and cardiac monitoring within 2 h of arrival and continuing for 24 h during hospitalization (26). In this context, a cluster randomized clinical trial evaluated whether a multifaceted quality improvement intervention could increase adherence to evidence-based therapies for the management of patients with acute ischemic stroke and transient ischemic attack in a hospital in Lima. However, the intervention did not achieve significant improvements in overall adherence (69).

### Community level: COVID-19

During the COVID-19 pandemic, there was an increase in published studies about ischemic stroke in Peru. There were five case reports of ischemic stroke in the context of COVID-19 infection in children, pregnant women, and adults (70–72) Across observational studies, several findings highlighted the relationship between stroke care and the COVID-19 pandemic in Peru. One analysis documented a relative reduction of 46.9 per 1,000 inhabitants in overall stroke volume, with an even sharper decline of 70.8 per 1,000 inhabitants in intravenous tPA procedures during the pandemic (73). Other comparisons of pre- and post-pandemic admissions found no statistically significant differences for acute ischemic stroke (*p* = 0.34) or transient ischemic attack (*p* = 0.46) (74). Patients with concomitant COVID-19 infection were noted to have higher baseline glucose, elevated neutrophil counts, reduced lymphocyte counts, and an increased neutrophil-to-lymphocyte ratio, alongside higher NIHSS scores at presentation (35). This might explain why stroke in the setting of COVID-19 was associated with high mortality and low rates of functional independence among survivors (24).

Limitations in access to healthcare were exacerbated during this period. During the lockdown, patients and their caregivers had limited mobilization around the city to arrive at a hospital. Also, they had trouble understanding the reorganization of stroke healthcare. Communication between caregivers and medical staff was reduced to fragmented telephone calls, no formal training was provided to caregivers, follow-up appointments were difficult to obtain due to an inefficient referral system, and telemedicine was implemented only to a limited extent because of restricted internet access and limited technological literacy among patients and caregivers (63).

### Policy and environmental level

There were eight studies that reported an analysis of nationwide estimated data regarding ischemic stroke frequency among general population, using secondary databases of healthcare systems registries. We observed a decrease in the age-standardized incidence rate of ischemic stroke from 60.28 in 1990 to 41.81 per 100,000 inhabitants in 2021 (75). Similar results obtained for disability-adjusted life years, YLD, and YLL related to ischemic stroke. However, during 2017, the age-standardized mortality rate was up to 853 per 100,000 inhabitants among individuals aged between 85 and 89 years old (76).

Preventive education is part of a key policy for stroke prevention. In this context, primary care interventions were developed using educational and digital tools which aimed to improve stroke knowledge and awareness in individuals with high cardiovascular risk. These interventions directly engaged individuals in the design and distribution of educational materials; however, it did not include clinical or impact evaluation of individuals after the intervention (77, 78).

There was only one study that reported environmental factors such as ambient particulate matter pollution, household air pollution from solid fuels, and lead exposure as the 3rd, 10th, and 13th, attributable causes to overall stroke, respectively (79). However, there were no studies that assessed impact of country policies' changes or implementations regarding ischemic stroke care.

## Discussion

### Summary of results

In our scoping review, we identified 119 primary studies of ischemic stroke in Peru, reflecting a gradual expansion of research activity over the past two decades. Most evidence came from isolated observational studies. Aggregate epidemiological estimates including the proportion of undetermined etiology (30%) and the thrombolysis rate (2.9%) are broadly consistent with studies from the region (80, 81). The scientific value of this review lies in mapping the context-specific drivers behind those numbers and in identifying where the research outputs fail to cover the actual burden and geography of disease in Peru.

Many challenges were identified, such as an incomplete work-up to identify non-usual etiologies in individual ischemic stroke reports, the very low thrombolysis rate and the absence of reports on mechanical thrombectomy for acute care, the lack of clinical results for innovative computer-based interventions, the heterogeneous qualitative descriptions of patient and caregivers' experiences during post-stroke care and health system access limitations, and few reports about policy interventions and environmental risk factors. Overall, these findings reveal a research ecosystem that, despite meaningful growth, is partially aligned with Peru's growing ischemic stroke burden, focused on already known descriptive epidemiology.

### Ischemic stroke epidemiology in Peru

Evidence regarding the epidemiology of ischemic stroke in Peru derives from local predominantly single-center studies with 79.1% originating from the capital city. Meanwhile, national-level estimates often depend on secondary data from healthcare systems, with reporting and methodological limitations (38, 82). The absence of population-based studies that consider Peru's cultural and geographical diversity likely resulted in an underestimation of the true burden of ischemic stroke and may bias policy decisions related to stroke prevention and care. Furthermore, analysis of ischemic stroke etiologies revealed that nearly one third of patients had an undetermined etiology, differing with a previous global meta-analysis (83). However, this result is within the range of other studies in Latin America (81), Brazil (84), Chile (85), Mexico (86). Thus, in high-income settings, cryptogenic stroke typically reflects residual uncertainty after exhaustive investigation, whereas in Peru and in Latin America, it may reflects a structural absence of the diagnostic tools required to complete that investigation, such as vascular imaging, prolonged cardiac monitoring, structural cardiac evaluations, or coagulopathy testing, contrary to recommendations from the 2021 AHA/ASA secondary prevention guidelines (87). These diagnostic gaps limit the identification of other specific etiologies with potential specific treatments to reduce the recurrence of ischemic stroke.

Regarding ischemic stroke risk factors, research findings in Peru align with well-established determinants such as high prevalences of hypertension, dyslipidemia, cardiovascular disease, hormonal contraceptive use, and aging (88). A few studies explored high blood viscosity in Andean populations living at high altitudes as a potential contributor for ischemic stroke (89, 90). Nonetheless, there was lack of information regarding other risk factors particularly relevant to the Peruvian context, including the high prevalence of intracranial arterial stenosis in native-Hispanic population, the effects of high-altitude residence, and the influence of socioeconomic conditions and cultural beliefs on chronic disease healthcare access and treatment adherence (91, 92). On the other hand, some studies identified ischemic stroke as a risk factor for depression and cognitive impairment in Peruvian population (39, 40). However, research has not yet explored the long-term social implications of stroke, effects on sexual health, or economic burden for households and the health system. These research gaps show the need for integrative, context-sensitive research agendas that can inform equitable and culturally adapted stroke prevention strategies in low- and middle-income settings.

### Evidence-based interventions for ischemic stroke in Peru

In Peru, interventional evidence remains scarce. While Peruvian patients participated in multicenter stroke prevention or therapeutic trials, the local and individualized data were unavailable. This is particularly relevant because the implementation and adherence of evidence-based recommendations for ischemic stroke in Peruvian centers were not achieved by a multifaceted quality improvement intervention during a randomized cluster trial (69). In parallel, local adherence to AHA/ASA measures has been suboptimal and thrombolysis rates historically low (9, 26). The thrombolysis rate ranged between 1.19% and 6.3% (9, 24, 31, 36, 93, 94), similar to rates reported in low- and middle-income countries around the world (95) and Latin America (81), like Brazil (96), while there are no reports of mechanical thrombectomy. This deficiency represents a system-level failure with directly actionable solution to multifactorial causes including delayed patient arrival outside the therapeutic window (9, 24), poor recognition of stroke symptoms (9, 36), lack of adequate pre-hospital notification and transport (9, 36), insufficient availability of CT and MRI scanners (24), and inadequate acute care flow (24). This is highlighted by a recently published study, not included in the present analysis, that reported an heterogenous thrombolysis rate between 7.7% to 26.3% across five Peruvian facilities from 2023 to 2025, where the highest rate was in a private center from Lima (68). Furthermore, there is still an urgent need for contextual adaptation in service delivery of well-known evidence-based interventions for stroke treatment (97, 98).

To address this critical gap in acute care and adherence, we propose a contextually adapted framework for the Peruvian health system. This framework requires establishing a national program that standardizes Code Stroke pathways across all levels of care and assures complete access to evidence-based interventions. Structurally, this entails dividing the country into geographical zones (central, north, south, and east) where third-level hospitals equipped with specialized stroke units must be interconnected for rapid decision-making, based on the 2023 Telemedicine Technical Guideline to expand tele-stroke networks beyond Lima. Finally, this program must incorporate rehabilitation centers and secondary prevention at the primary and secondary levels, alongside quality monitoring and multicenter research networks, ensuring actions align with global recommendations and system guidance for middle-income nations (7, 99, 100).

### Caregivers and rehabilitation in post-stroke patients in Peru

Findings from both quantitative and qualitative studies highlight the gaps in post-stroke care in Peru, particularly regarding assistance and training for caregivers, rehabilitation programs, and access to palliative care. The post-stroke care continuum is fragmented, marked by systemic barriers to healthcare access (11, 24, 101), including financial constraints, affecting low-income populations (102), the absence of structured palliative care training programs (60, 103), insufficient institutional support for caregivers (61, 63, 102). After hospital discharge, stroke survivors were frequently discharged to their home despite needing specialized nursing or palliative support, transferring the burden of care to family members who often lack both financial and technical resources (60, 63). This lack of continuity of care forces caregivers to assume complex roles without adequate technical or economic resources, severely interrupting the rehabilitation process.

This reality exposes profound disparities when compared with high-income countries, where the access to rehabilitation program is about 66% (104), higher compared to low- and middle-income countries settings, such as Peru where there is not a national palliative care hospital, program or policy (11, 63, 103). Strengthening post-stroke care in Peru and similar low- and middle-income countries requires the implementation of multicomponent interventions that integrate rehabilitation, palliative care, and psychological support. Such an approach should prioritize community-based delivery models and caregiver training to enhance independence in activities of daily living, reduce caregiver burden, and ultimately improve long-term outcomes after stroke.

### Limitations and strengths

This scoping review is limited by substantial heterogeneity in study designs, populations, outcomes, and reporting, which precluded quantitative synthesis and limited direct comparability across studies. Also, the exclusion of cerebral venous thrombosis means that our estimates should be interpreted as representing arterial ischemic stroke rather than the broader burden of all ischemic cerebrovascular disease. This may be particularly relevant in younger women, given the association of cerebral venous thrombosis with hormonal exposure, pregnancy, and the puerperium (105). The classification following a socio-ecological approach involved some degree of subjective judgment, as determinants may overlap across socio-ecological levels despite the use of consensus-based classification. Also, there is missing information among studies for critical outcomes such as disability and mortality, and how these present in relation to other clinical and imaging characteristics. The literature was heavily concentrated in Lima and in facility-based secondary data, introducing geographic and selection biases and likely underestimating stroke burden in rural, Andean, and Amazonian populations. Publication bias (unpublished local reports and grey literature) and the paucity of large and randomized trials further limit the generalizability of some findings. Finaly, despite that most of the included reports on interventional rehabilitation technologies and community interventions came from conference abstracts lacking proper peer-review and validity for applicability, we included them because of the limited number of originally published interventional studies.

Scoping reviews addressing specific aspects of stroke care have been conducted in other Latin American countries^(^106–108). However, we did not identify a directly comparable national evidence-mapping study integrating epidemiological, clinical, organizational, community, and policy evidence within a socio-ecological framework. This review provides the most comprehensive mapping to date of published empirical evidence on ischemic stroke in Peru. We used a sensitive search strategy to include and integrate different study designs including observational, interventional, qualitative, and case-report literature. Use of explicit scoping methodology and inclusion of implementation-relevant outcomes (timing, thrombolysis rates, caregiver experiences, and adherence to performance measures) enhances the review's utility for clinicians, health-system planners and researchers.

## Conclusion

Evidence on ischemic stroke in Peru is concentrated in urban tertiary centers, heterogeneous, and limited by frequent incomplete etiologic workups. Aggregated epidemiological estimates are broadly consistent with regional reports, such as high proportions of undetermined etiology and very low reperfusion treatment access. However, the identified context-specific causes for these estimates were diagnostic infrastructure deficits, absence of acute care pathways, and fragmented post-stroke support. Major gaps persist in rural and high-altitude settings, rehabilitation, caregiver support, and palliative care. To improve outcomes, Peru will require a nationally coordinated, regionally distributed stroke program that standardizes acute pathways, strengthens diagnostic capacity, and scales community-based rehabilitation, caregiver training, mental-health, and palliative services.

## Data Availability

The original contributions presented in the study are included in the article/Supplementary Material, further inquiries can be directed to the corresponding author.
