# Supplementary material for: Ischemic stroke in Peru: a scoping review of trends, outcomes, and evidence gaps
Source: Front Epidemiol. 2026 Aug 28;6:1926861. doi: 10.3389/fepid.2026.1926861 (PMC13562258; doi:10.3389/fepid.2026.1926861)
Supplement: Supplementary file 1 [file Table1.docx]

**Table S1: Preferred Reporting Items for Systematic reviews and Meta-Analyses extension for Scoping Reviews (PRISMA-ScR) Checklist**

| **SECTION** | **ITEM** | **PRISMA-ScR CHECKLIST ITEM** | **REPORTED ON PAGE #** |
| --- | --- | --- | --- |
| **TITLE** | | | |
| Title | 1 | Identify the report as a scoping review. | Click here to enter text. |
| **ABSTRACT** | | | |
| Structured summary | 2 | Provide a structured summary that includes (as applicable): background, objectives, eligibility criteria, sources of evidence, charting methods, results, and conclusions that relate to the review questions and objectives. | Click here to enter text. |
| **INTRODUCTION** | | | |
| Rationale | 3 | Describe the rationale for the review in the context of what is already known. Explain why the review questions/objectives lend themselves to a scoping review approach. | Click here to enter text. |
| Objectives | 4 | Provide an explicit statement of the questions and objectives being addressed with reference to their key elements (e.g., population or participants, concepts, and context) or other relevant key elements used to conceptualize the review questions and/or objectives. | Click here to enter text. |
| **METHODS** | | | |
| Protocol and registration | 5 | Indicate whether a review protocol exists; state if and where it can be accessed (e.g., a Web address); and if available, provide registration information, including the registration number. | Click here to enter text. |
| Eligibility criteria | 6 | Specify characteristics of the sources of evidence used as eligibility criteria (e.g., years considered, language, and publication status), and provide a rationale. | Click here to enter text. |
| Information sources* | 7 | Describe all information sources in the search (e.g., databases with dates of coverage and contact with authors to identify additional sources), as well as the date the most recent search was executed. | Click here to enter text. |
| Search | 8 | Present the full electronic search strategy for at least 1 database, including any limits used, such that it could be repeated. | Click here to enter text. |
| Selection of sources of evidence† | 9 | State the process for selecting sources of evidence (i.e., screening and eligibility) included in the scoping review. | Click here to enter text. |
| Data charting process‡ | 10 | Describe the methods of charting data from the included sources of evidence (e.g., calibrated forms or forms that have been tested by the team before their use, and whether data charting was done independently or in duplicate) and any processes for obtaining and confirming data from investigators. | Click here to enter text. |
| Data items | 11 | List and define all variables for which data were sought and any assumptions and simplifications made. | Click here to enter text. |
| Critical appraisal of individual sources of evidence§ | 12 | If done, provide a rationale for conducting a critical appraisal of included sources of evidence; describe the methods used and how this information was used in any data synthesis (if appropriate). | Click here to enter text. |
| Synthesis of results | 13 | Describe the methods of handling and summarizing the data that were charted. | Click here to enter text. |
| **RESULTS** | | | |
| Selection of sources of evidence | 14 | Give numbers of sources of evidence screened, assessed for eligibility, and included in the review, with reasons for exclusions at each stage, ideally using a flow diagram. | Click here to enter text. |
| Characteristics of sources of evidence | 15 | For each source of evidence, present characteristics for which data were charted and provide the citations. | Click here to enter text. |
| Critical appraisal within sources of evidence | 16 | If done, present data on critical appraisal of included sources of evidence (see item 12). | Click here to enter text. |
| Results of individual sources of evidence | 17 | For each included source of evidence, present the relevant data that were charted that relate to the review questions and objectives. | Click here to enter text. |
| Synthesis of results | 18 | Summarize and/or present the charting results as they relate to the review questions and objectives. | Click here to enter text. |
| **DISCUSSION** | | | |
| Summary of evidence | 19 | Summarize the main results (including an overview of concepts, themes, and types of evidence available), link to the review questions and objectives, and consider the relevance to key groups. | Click here to enter text. |
| Limitations | 20 | Discuss the limitations of the scoping review process. | Click here to enter text. |
| Conclusions | 21 | Provide a general interpretation of the results with respect to the review questions and objectives, as well as potential implications and/or next steps. | Click here to enter text. |
| **FUNDING** | | | |
| Funding | 22 | Describe sources of funding for the included sources of evidence, as well as sources of funding for the scoping review. Describe the role of the funders of the scoping review. | Click here to enter text. |

JBI = Joanna Briggs Institute; PRISMA-ScR = Preferred Reporting Items for Systematic reviews and Meta-Analyses extension for Scoping Reviews.

* Where *sources of evidence* (see second footnote) are compiled from, such as bibliographic databases, social media platforms, and Web sites.

† A more inclusive/heterogeneous term used to account for the different types of evidence or data sources (e.g., quantitative and/or qualitative research, expert opinion, and policy documents) that may be eligible in a scoping review as opposed to only studies. This is not to be confused with *information sources* (see first footnote).

‡ The frameworks by Arksey and O’Malley (6) and Levac and colleagues (7) and the JBI guidance (4, 5) refer to the process of data extraction in a scoping review as data charting*.*

§ The process of systematically examining research evidence to assess its validity, results, and relevance before using it to inform a decision. This term is used for items 12 and 19 instead of "risk of bias" (which is more applicable to systematic reviews of interventions) to include and acknowledge the various sources of evidence that may be used in a scoping review (e.g., quantitative and/or qualitative research, expert opinion, and policy document).

*From:* Tricco AC, Lillie E, Zarin W, O'Brien KK, Colquhoun H, Levac D, et al. PRISMA Extension for Scoping Reviews (PRISMAScR): Checklist and Explanation. Ann Intern Med. 2018;169:467–473. [doi: 10.7326/M18-0850](http://annals.org/aim/fullarticle/2700389/prisma-extension-scoping-reviews-prisma-scr-checklist-explanation).

**Table S2: Search strategies**

| **Database** | **Search strategy** |
| --- | --- |
| Scopus | TITLE-ABS (stroke* OR apoplex* OR "Acute Ischemic attack*" OR "Acute Ischaemic attack*" OR "transient ischemic attack*" OR "transient ischaemic attack*" OR cerebrovascular OR ( ( cerebr* OR brain OR cerebellum OR cerebrum ) NEAR/1 ( vascul* OR ischemi* OR ischaemi* OR infarct* ) ) ) AND (AFFILCOUNTRY ( Peru) OR TITLE-ABS(Peru*) ) |
| Web of Science | (TI=( *stroke* OR apoplex* OR "Acute Ischemic attack*" OR "Acute Ischaemic attack*" OR "transient ischemic attack*" OR "transient ischaemic attack*" OR cerebrovascular OR ( ( cerebr* OR brain OR cerebellum OR cerebrum ) NEAR/1 ( vascul* OR ischemi* OR ischaemi* OR infarct* ) ) )  OR  AB=(stroke* OR apoplex* OR "Acute Ischemic attack*" OR "Acute Ischaemic attack*" OR "transient ischemic attack*" OR "transient ischaemic attack*" OR cerebrovascular OR ( ( cerebr* OR brain OR cerebellum OR cerebrum ) NEAR/1 ( vascul* OR ischemi* OR ischaemi* OR infarct* ) ) ) )  AND  ( CU=(Peru) OR TI=(Peru*) OR AB=(Peru*)) |
| Scielo | ( TI=( *stroke* OR apoplex* OR "Acute Ischemic attack*" OR "Acute Ischaemic attack*" OR ictus OR "transient ischemic attack*" OR "transient ischaemic attack*" OR “Ataque isquemico transitorio” OR cerebrovascular OR ( ( cerebr* OR brain OR cerebellum OR cerebrum) NEAR/1 ( vascul* OR ischemi* OR ischaemi* OR infarct* OR infarto OR isquemic*) ) )  OR  AB=(stroke* OR apoplex* OR "Acute Ischemic attack*" OR "Acute Ischaemic attack*" OR ictus OR "transient ischemic attack*" OR "transient ischaemic attack*" OR “Ataque isquemico transitorio” OR cerebrovascular OR ( ( cerebr* OR brain OR cerebellum OR cerebrum) NEAR/1 ( vascul* OR ischemi* OR ischaemi* OR infarct* OR infarto OR isquemic*) ) ) )  AND  ( CU=(Peru) OR TI=(Peru*) OR AB=(Peru*)) |
| Pubmed | (stroke* OR apoplex* OR "Acute Ischemic attack*" OR "Acute Ischaemic attack*" OR "transient ischemic attack*" OR "transient ischaemic attack*" OR cerebrovascular OR "Cerebral Infarct"[tiab:~1] OR "Cerebral Ischaemic"[tiab:~1] OR "Cerebral Ischemic"[tiab:~1] OR "Cerebral Vascular"[tiab:~1] OR "Brain Infarct"[tiab:~1] OR "Brain Ischaemic"[tiab:~1] OR "Brain Ischemic"[tiab:~1] OR "Brain Vascular"[tiab:~1] OR "Cerebellum Infarct"[tiab:~1] OR "Cerebellum Ischaemic"[tiab:~1] OR "Cerebellum Ischemic"[tiab:~1] OR "Cerebellum Vascular"[tiab:~1] OR "Cerebrum Infarct"[tiab:~1] OR "Cerebrum Ischaemic"[tiab:~1] OR "Cerebrum Ischemic"[tiab:~1] OR "Cerebrum Vascular"[tiab:~1]) AND (Peru OR Peruvian*) |

**Table S3: Excluded reports during full-text assessment and reasons**

| **Title** | **Year** | **Reason to exclude** |
| --- | --- | --- |
| LATIN AMERICAN STROKE REGISTRY | 2018 | Includes population from Peru, but there are no individual results from them |
| Sex Differences Among Participants in the Latin American Stroke Registry | 2020 | Includes population from Peru, but there are no individual results from them |
| Ticagrelor Versus Clopidogrel in Patients With STEMI Treated With Fibrinolysis TREAT Trial | 2019 | Includes population from Peru, but there are no individual results from them |
| Ticagrelor vs clopidogrel after fibrinolytic therapy in patients with st-elevation myocardial infarction a randomized clinical trial | 2018 | Includes population from Peru, but there are no individual results from them |
| Effect of Alirocumab on Lipoprotein(a) and Cardiovascular Risk After Acute Coronary Syndrome | 2020 | Includes population from Peru, but there are no individual results from them |
| Long-Term Use of Ticagrelor in Patients with Prior Myocardial Infarction | 2015 | Includes population from Peru, but there are no individual results from them |
| Risk of stroke and other adverse outcomes in patients with perioperative atrial fibrillation 1 year after non-cardiac surgery | 2020 | Includes population from Peru, but there are no individual results from them |
| Anticoagulant therapy and outcomes in patients with prior or acute heart failure and acute coronary syndromes: Insights from the APixaban for PRevention of Acute ISchemic Events 2 trial | 2015 | Includes population from Peru, but there are no individual results from them |
| Stroke Care and Application of Thrombolysis in Ibero-America Report From the SITS-SIECV Ibero-American Stroke Register | 2019 | Includes population from Peru, but there are no individual results from them |
| Effectiveness and safety of dabigatran in Latin American patients with atrial fibrillation: Two years follow up results from GLORIA-AF registry | 2020 | Includes population from Peru, but there are no individual results from them |
| C-Reactive Protein as a Prognostic Marker After Lacunar Stroke Levels of Inflammatory Markers in the Treatment of Stroke Study | 2014 | Includes population from Peru, but there are no individual results from them |
| Global, regional, and national burden of neurological disorders during 1990–2015: a systematic analysis for the Global Burden of Disease Study 2015 | 2017 | Includes population from Peru, but there are no individual results from them |
| Global, regional, and national burden of neurological disorders, 1990-2016: a systematic analysis for the Global Burden of Disease Study 2016 | 2019 | Includes population from Peru, but there are no individual results from them |
| Cerebral Vascular Disease in patients with COVID-19: Preliminary report in 3 Latin American cities | 2022 | Includes population from Peru, but there are no individual results from them |
| Comparison of fatal or irreversible events with extended-duration betrixaban versus standard dose enoxaparin in acutely Ill medical patients: An APEX trial substudy | 2017 | Includes population from Peru, but there are no individual results from them |
| Edoxaban versus Warfarin in Patients with Atrial Fibrillation | 2013 | Includes population from Peru, but there are no individual results from them |
| Transcranial laser therapy in acute stroke treatment: Results of neurothera effectiveness and safety trial 3, a phase III clinical end point device trial | 2014 | Includes population from Peru, but there are no individual results from them |
| Urinary Sodium and Potassium, and Risk of Ischemic and Hemorrhagic Stroke (INTERSTROKE): A Case-Control Study | 2021 | Includes population from Peru, but there are no individual results from them |
| Alirocumab in Patients With Polyvascular Disease and Recent Acute Coronary Syndrome ODYSSEY OUTCOMES Trial | 2019 | Includes population from Peru, but there are no individual results from them |
| Endemic cardiovascular diseases of the poorest billion | 2016 | Includes population from Peru, but there are no individual results from them |
| Global, regional, and national disability-adjusted life-years (DALYs) for 359 diseases and injuries and healthy life expectancy (HALE) for 195 countries and territories, 1990-2017: a systematic analysis for the Global Burden of Disease Study 2017 | 2018 | Includes population from Peru, but there are no individual results from them |
| Infrared laser therapy for ischemic stroke: A new treatment strategy results of the NeuroThera effectiveness and Safety Trial-1 (NEST-1) | 2007 | Includes population from Peru, but there are no individual results from them |
| Practice patterns and outcomes after stroke across countries at different economic levels (INTERSTROKE): an international observational study | 2018 | Includes population from Peru, but there are no individual results from them |
| An international cluster-randomized quality improvement trial to increase the adherence to evidence-based therapies for acute ischemic stroke and transient ischemic attack patients: Rationale and design of the BRIDGE STROKE Trial | 2019 | Includes population from Peru, but there are no individual results from them |
| Safety and Outcome of Revascularization Treatment in Patients With Acute Ischemic Stroke and COVID-19: The Global COVID-19 Stroke Registry | 2023 | Includes population from Peru, but there are no individual results from them |
| Perioperative covert stroke in patients undergoing non-cardiac surgery (NeuroVISION): a prospective cohort study | 2019 | Includes population from Peru, but there are no individual results from them |
| Global Impact of the COVID-19 Pandemic on Stroke Volumes and Cerebrovascular Events: A 1-Year Follow-up | 2023 | Includes population from Peru, but there are no individual results from them |
| Global impact of COVID-19 on stroke care | 2021 | Includes population from Peru, but there are no individual results from them |
| Variations in knowledge, awareness and treatment of hypertension and stroke risk by country income level | 2021 | Includes population from Peru, but there are no individual results from them |
| Global and regional effects of potentially modifiable risk factors associated with acute stroke in 32 countries (INTERSTROKE): a case-control study | 2016 | Includes population from Peru, but there are no individual results from them |
| Association of Lipids, Lipoproteins, and Apolipoproteins with Stroke Subtypes in an International Case Control Study (INTERSTROKE) | 2022 | Includes population from Peru, but there are no individual results from them |
| Burden of disease due to stroke in Latin America: A systematic analysis of the global burden disease study 2017 | 2019 | Includes population from Peru, but there are no individual results from them |
| Burden of disease due to stroke and risk factors in Latin America: an analysis of the Global Burden Disease Study | 2020 | Includes population from Peru, but there are no individual results from them |
| Achieved blood pressures in the secondary prevention of small subcortical strokes (SPS3) study: challenges and lessons learned | 2014 | Includes population from Peru, but there are no individual results from them |
| Cause of death and predictors of all-cause mortality in anticoagulated patients with nonvalvular atrial fibrillation: Data from ROCKET AF | 2015 | Includes population from Peru, but there are no individual results from them |
| Stroke-Related Hospital Admissions During Covid-19 Pandemic in the Latin American Stroke Registry (LASE) | 2021 | Includes population from Peru, but there are no individual results from them |
| Holmes tremor: Clinical description, lesion localization, and treatment in a series of 29 cases | 2016 | Includes population from Peru, but there are no individual results from them |
| Effects of alirocumab on cardiovascular and metabolic outcomes after acute coronary syndrome in patients with or without diabetes: a prespecified analysis of the ODYSSEY OUTCOMES randomised controlled trial | 2019 | Includes population from Peru, but there are no individual results from them |
| Association of Psychosocial Stress With Risk of Acute Stroke | 2022 | Includes population from Peru, but there are no individual results from them |
| Risk Categorization Using New American College of Cardiology/American Heart Association Guidelines for Cholesterol Management and Its Relation to Alirocumab Treatment Following Acute Coronary Syndromes | 2019 | Includes population from Peru, but there are no individual results from them |
| One-year Outcomes after Discharge from Noncardiac Surgery and Association between Predischarge Complications and Death after Discharge: Analysis of the VISION Prospective Cohort Study | 2024 | Includes population from Peru, but there are no individual results from them |
| Outcomes With Edoxaban Versus Warfarin in Patients With Previous Cerebrovascular Events Findings From ENGAGE AF-TIMI 48 (Effective Anticoagulation With Factor Xa Next Generation in Atrial Fibrillation-Thrombolysis in Myocardial Infarction 48) | 2016 | Includes population from Peru, but there are no individual results from them |
| Global Burden of Cardiovascular Diseases and Risk Factors, 1990-2019: Update From the GBD 2019 Study | 2020 | Includes population from Peru, but there are no individual results from them |
| Efficacy and safety of non-immersive virtual reality exercising in stroke rehabilitation (EVREST): a randomised, multicentre, single-blind, controlled trial | 2016 | Includes population from Peru, but there are no individual results from them |
| Alirocumab and cardiovascular outcomes after acute coronary syndrome | 2018 | Includes population from Peru, but there are no individual results from them |
| Saxagliptin and Cardiovascular Outcomes in Patients with Type 2 Diabetes Mellitus | 2013 | Includes population from Peru, but there are no individual results from them |
| Effect of alirocumab on cardiovascular outcomes after acute coronary syndromes according to age: an ODYSSEY OUTCOMES trial analysis | 2020 | Includes population from Peru, but there are no individual results from them |
| Renal Impairment and Risk of Acute Stroke: The INTERSTROKE Study | 2021 | Includes population from Peru, but there are no individual results from them |
| Responsiveness of the Reaching Performance Scale for Stroke | 2023 | Includes population from Peru, but there are no individual results from them |
| Alirocumab Reduces Total Hospitalizations and Increases Days Alive and Out of Hospital in the ODYSSEY OUTCOMES Trial | 2019 | Includes population from Peru, but there are no individual results from them |
| Alirocumab Reduces Total Nonfatal Cardiovascular and Fatal Events The ODYSSEY OUTCOMES Trial | 2019 | Includes population from Peru, but there are no individual results from them |
| IV thrombolysis and general stroke care in Latin America - Report from the SIECV-SITS Iberoamerican Stroke Register | 2015 | Includes population from Peru, but there are no individual results from them |
| Lipoprotein-associated phospholipase A2 activity is a marker of risk but not a useful target for treatment in patients with stable coronary heart disease | 2016 | Includes population from Peru, but there are no individual results from them |
| Impact of Spontaneous Extracranial Bleeding Events on Health State Utility in Patients with Atrial Fibrillation: Results from the ENGAGE AF-TIMI 48 Trial | 2017 | Includes population from Peru, but there are no individual results from them |
| Blood Pressure After Recent Stroke: Baseline Findings From the Secondary Prevention of Small Subcortical Strokes Trial | 2013 | Includes population from Peru, but there are no individual results from them |
| Clinical Features and Racial/Ethnic Differences Among the 3020 Participants in the Secondary Prevention of Small Subcortical Strokes (SPS3) Trial | 2013 | Includes population from Peru, but there are no individual results from them |
| Darapladib for preventing ischemic events in stable coronary heart disease | 2014 | Includes population from Peru, but there are no individual results from them |
| Twelve-month safety of intravitreal injections of bevacizumab (Avastin®):: results of the Pan-American Collaborative Retina Study Group (PACORES) | 2008 | Includes population from Peru, but there are no individual results from them |
| Empagliflozin and Cerebrovascular Events in Patients With Type 2 Diabetes Mellitus at High Cardiovascular Risk | 2017 | Includes population from Peru, but there are no individual results from them |
| Empagliflozin, Cardiovascular Outcomes, and Mortality in Type 2 Diabetes | 2015 | Includes population from Peru, but there are no individual results from them |
| Effectiveness and Safety of Transcranial Laser Therapy for Acute Ischemic Stroke | 2009 | Includes population from Peru, but there are no individual results from them |
| Role of mHealth in overcoming the occurrence of post-stroke depression | 2018 | It is not a primary study |
| Addressing post-stroke care in rural areas with Peru as a case study. Placing emphasis on evidence-based pragmatism | 2017 | It is not a primary study |
| Expanding antiplatelet use for patients with stroke: The case for stroke of unknown type | 2014 | It is not a primary study |
| Cuidadores informales de pacientes con enfermedades cerebrovasculares | 2014 | It is not a primary study |
| Posterior reversible encephalopathy syndrome in a child with severe multisystem inflammatory syndrome due to COVID-19 | 2022 | There is no information on stroke (neither population, nor variables, nor outcomes) |
| Global and National Burden of Diseases and Injuries Among Children and Adolescents Between 1990 and 2013: Findings From the Global Burden of Disease 2013 Study | 2016 | There is no information on stroke (neither population, nor variables, nor outcomes) |
| Neuroimaging manifestations in children with SARS-CoV-2 infection: a multinational, multicentre collaborative study | 2021 | There is no information on stroke (neither population, nor variables, nor outcomes) |
| Application of Machine Learning for the Prediction of Strokes in Peru | 2022 | There is no information on stroke (neither population, nor variables, nor outcomes) |
| Global Impact of the COVID-19 Pandemic on Cerebral Venous Thrombosis and Mortality | 2022 | There is no information on stroke (neither population, nor variables, nor outcomes) |
| Angiotensin receptor neprilysin inhibition compared with enalapril on the risk of clinical progression in surviving patients with heart failure | 2015 | There is no information on stroke (neither population, nor variables, nor outcomes) |
| Visual vignette. Pituitary apoplexy | 2011 | There is no information on stroke (neither population, nor variables, nor outcomes) |
| Apoplejía pituitaria: Reporte de dos casos | 2007 | There is no information on stroke (neither population, nor variables, nor outcomes) |
| Apoplejía pituitaria con parálisis del III par craneal: Reporte de caso | 2011 | There is no information on stroke (neither population, nor variables, nor outcomes) |
| Pituitary apoplexy. A case report | 2013 | There is no information on stroke (neither population, nor variables, nor outcomes) |
| Enfermedad de Moyamoya: reporte de un caso | 2015 | There is no information on stroke (neither population, nor variables, nor outcomes) |
| Prevalence of diabetes and risk factors for coronary heart disease and stroke in Peruvian mestizo population | 1997 | There is no information on stroke (neither population, nor variables, nor outcomes) |
| Cardiovascular outcomes of type 2 diabetic patients treated with DPP-4 inhibitors versus sulphonylureas as add-on to metformin in clinical practice | 2021 | Does not include population in Peru (Total from another country) |
| Stroke and pulmonary thromboembolism after a long flight | 2005 | Does not include population in Peru (Total from another country) |
| Edoxaban Versus Warfarin in Latin American Patients With Atrial Fibrillation: The ENGAGE AF-TIMI 48 Trial | 2018 | Does not include population in Peru (Total from another country) |
| Amyloid Transthyretin Cardiomyopathy in Elderly Patients With Aortic Stenosis Undergoing Transcatheter Aortic Valve Implantation | 2023 | Does not include population in Peru (Total from another country) |
| The impact of stroke in Latin America: A longitudinal analysis of the burden of disease and risk factors (1990-2019) | 2021 | Does not include population in Peru (Total from another country) |
| Cerebrovascular Events in 21 105 Patients With Atrial Fibrillation Randomized to Edoxaban Versus Warfarin Effective Anticoagulation With Factor Xa Next Generation in Atrial Fibrillation-Thrombolysis in Myocardial Infarction 48 | 2014 | Does not include population in Peru (Total from another country) |
| Bilateral Vertebral Artery Dissection: A Case Report with Literature Review | 2020 | Does not include population in Peru (Total from another country) |
| Effects of Alirocumab on Cardiovascular Events After Coronary Bypass Surgery | 2019 | Does not include population in Peru (Total from another country) |
| The association between metabolic syndrome and major adverse cardiac and cerebrovascular events in patients with acute coronary syndrome undergoing percutaneous coronary intervention | 2024 | Does not include population in Peru (Total from another country) |
| Concerning Rise In Young-onset Stroke Hospitalizations And Non-improving Outcomes In Patients From Low Household Income Quartile: A National Perspective A Decade Apart (2007 Vs. 2017) | 2022 | Does not include population in Peru (Total from another country) |
| Low dose intravenous cangrelor versus glycoprotein IIb/IIIa inhibitors in endovascular treatment of tandem lesions | 2023 | Does not include population in Peru (Total from another country) |
| Empagliflozin and Cardiovascular Outcomes in Asian Patients With Type 2 Diabetes and Established Cardiovascular Disease - Results From EMPA-REG OUTCOME® | 2017 | Does not include population in Peru (Total from another country) |
| Global health, global surgery and mass casualties. I. Rationale for integrated mass casualty centres | 2019 | Does not include population in Peru (Total from another country) |
| Subtypes of Ischemic Stroke in the Young | 2013 | Does not include population in Peru (Total from another country) |
| The impact of body mass index on oncoplastic breast surgery: A multicenter analysis | 2023 | Does not include population in Peru (Total from another country) |
| Stroke and Mortality Risk in Patients With Various Patterns of Atrial Fibrillation Results From the ENGAGE AF-TIMI 48 Trial (Effective Anticoagulation With Factor Xa Next Generation in Atrial Fibrillation-Thrombolysis in Myocardial Infarction 48) | 2017 | Does not include population in Peru (Total from another country) |
| Efficacy and safety of ticagrelor for long-term secondary prevention of atherothrombotic events in relation to renal function: Insights from the PEGASUS-TIMI 54 trial | 2016 | Does not include population in Peru (Total from another country) |
| The Mystery of a Unilateral Headache Ultimately Diagnosed as Moyamoya Disease | 2022 | Does not include population in Peru (Total from another country) |
| Transition of care for the elderly after cerebrovascular accidents - From hospital to the home | 2013 | Does not include population in Peru (Total from another country) |
| Endovascular strategies for stroke management after subacute in-stent thrombosis | 2020 | Does not include population in Peru (Total from another country) |
| Safe and Efficacious Use of 1-Month Triple Therapy in Patients with Atrial Fibrillation and High Bleeding Risk Undergoing PCI | 2019 | Does not include population in Peru (Total from another country) |
| Association of Diabetes Mellitus and Its Types with In-Hospital Management and Outcomes of Patients with Acute Myocardial Infarction | 2023 | Does not include population in Peru (Total from another country) |
| [Impact of pretreatment with P2Y12 inhibitors in patients with acute coronary syndromes without ST elevation. Analysis of 2 multicenter registries] | 2023 | Does not include population in Peru (Total from another country) |
| Neurological diseases in Peru: a systematic analysis of the global burden disease study | 2020 | It does not differentiate between ischemic or hemorrhagic stroke |
| Medicación potencialmente inadecuada como factor de riesgo para el delírium en adultos mayores, en un servicio de emergencia | 2015 | It does not differentiate between ischemic or hemorrhagic stroke |
| Mortality trend due to cerebrovascular disease in the Ministry of Health of Peru, 2005-2015 | 2019 | It does not differentiate between ischemic or hemorrhagic stroke |
| Convulsive Status Epilepticus in a Cohort of Patients from a Peruvian Academic Hospital | 2021 | It does not differentiate between ischemic or hemorrhagic stroke |
| High prevalence of cardiovascular disease in end-stage kidney disease patients ongoing hemodialysis in Peru: Why should we care about it? | 2015 | It does not differentiate between ischemic or hemorrhagic stroke |
| Prevalence of Pragmatically Defined High CV Risk and its Correlates in LMIC A Report From 10 LMIC Areas in Africa, Asia, and South America | 2016 | It does not differentiate between ischemic or hemorrhagic stroke |
| Predictors of potential adverse reactions and drug-drug interactions in post-stroke patients in Peru | 2022 | It does not differentiate between ischemic or hemorrhagic stroke |
| Dislipidemia como factor de riesgo para enfermedad cerebrovascular: estudio de casos y controles | 2016 | It does not differentiate between ischemic or hemorrhagic stroke |
| Stroke in the setting of COVID-19 infection among patients attending a public hospital in Lima, Peru | 2021 | It does not differentiate between ischemic or hemorrhagic stroke |
| Socioeconomic Factors and All Cause and Cause-Specific Mortality among Older People in Latin America, India, and China: A Population-Based Cohort Study | 2012 | It does not differentiate between ischemic or hemorrhagic stroke |
| Prevalence of stroke and related burden among older people living in Latin America, India and China | 2011 | It does not differentiate between ischemic or hemorrhagic stroke |
| Factores clínicos y epidemiológicos relacionados a mortalidad en pacientes con estatus epiléptico en un hospital de Lima: una serie comparativa de casos | 2016 | It does not differentiate between ischemic or hemorrhagic stroke |
| Global, regional, and national disability-adjusted life-years (DALYs) for 333 diseases and injuries and healthy life expectancy (HALE) for 195 countries and territories, 1990-2016: A systematic analysis for the Global Burden of Disease Study 2016 | 2017 | It does not differentiate between ischemic or hemorrhagic stroke |
| Chronic diseases among older people and co-resident psychological morbidity: a 10/66 Dementia Research Group population-based survey | 2011 | It does not differentiate between ischemic or hemorrhagic stroke |
| ASSOCIATION OF STROKE WITH HIGHER BLOOD VISCOSITY IN POPULATION LIVING AT HIGH ALTITUDE (CUSCO 3399 MASL) | 2021 | It does not differentiate between ischemic or hemorrhagic stroke |
| Age Does Not Affect Metoprolol's Effect on Perioperative Outcomes (From the POISE Database) | 2018 | It does not differentiate between ischemic or hemorrhagic stroke |
| Prevalence of stroke at high altitude (3380 m) in Cuzco, a town of Peru a population-based study | 1995 | It does not differentiate between ischemic or hemorrhagic stroke |
| Associations between Depression, Depressive Symptoms, and Incidence of Dementia in Latin America: A 10/66 Dementia Research Group Study | 2019 | It does not differentiate between ischemic or hemorrhagic stroke |
| Population-based stroke incidence estimates in Peru: Exploratory results from the CRONICAS cohort study | 2022 | It does not differentiate between ischemic or hemorrhagic stroke |
| Low HDL cholesterol as a cardiovascular risk factor in rural, urban, and rural-urban migrants: PERU MIGRANT cohort study | 2016 | It does not differentiate between ischemic or hemorrhagic stroke |
| Características epidemiológicas de la neumonía intrahospitalaria en un servicio de medicina interna del Hospital Guillermo Almenara Irigoyen de Lima, Perú | 2016 | It does not differentiate between ischemic or hemorrhagic stroke |
| Seguimiento de pacientes con pie diabético en un hospital de alta complejidad del norte del Perú | 2021 | It does not differentiate between ischemic or hemorrhagic stroke |
| Factores de riesgo asociados a complicaciones intrahospitalarias, en adultos mayores del Hospital Nacional Edgardo Rebagliati Martins Lima, 2010 | 2015 | It does not differentiate between ischemic or hemorrhagic stroke |
| Assessing sexual dysfunction among stroke survivors and barriers to address this issue by physicians at a Latin American reference hospital | 2021 | It does not differentiate between ischemic or hemorrhagic stroke |
| Infecciones intrahospitalarias del tracto urinario en servicios críticos de un hospital público de Chiclayo, Perú (2009-2014) | 2016 | It does not differentiate between ischemic or hemorrhagic stroke |
| Priorities to reduce the burden of stroke in Latin American countries | 2019 | It does not differentiate between ischemic or hemorrhagic stroke |
| Lifestyle Risk Factors Associated with Stroke in Patients with Type 2 Diabetes Mellitus: A Case-control Study | 2023 | It does not differentiate between ischemic or hemorrhagic stroke |
| Frecuencia de casos de úlceras de presión y factores asociados a su desarrollo en adultos mayores hospitalizados en servicios de Medicina de un hospital general | 2009 | It does not differentiate between ischemic or hemorrhagic stroke |
| Financial impact of smoking on health systems in Latin America: A study of seven countries and extrapolation to the regional level | 2016 | It does not differentiate between ischemic or hemorrhagic stroke |
| Asociación entre la necesidad de cuidados paliativos y enfermedades crónicas en pacientes atendidos en un hospital peruano | 2021 | It does not differentiate between ischemic or hemorrhagic stroke |
| Psychological Impact of the Pandemic on Ambulatory Cardiometabolic Patients Without Evidence of SARS-CoV-2 Infection. The CorCOVID Latam Psy Study | 2021 | It does not differentiate between ischemic or hemorrhagic stroke |
| Cardiovascular risk factors for heart disease and stroke in women by age and time since menopause, in seven Latin American cities: The CARMELA study | 2008 | It does not differentiate between ischemic or hemorrhagic stroke |
| Síndrome cardiorrenal tipo 1 en la unidad de cuidados intensivos coronarios del Hospital Nacional Arzobispo Loayza | 2015 | It does not differentiate between ischemic or hemorrhagic stroke |
| Intrinsic capacity and its associations with incident dependence and mortality in 10/66 Dementia Research Group studies in Latin America, India, and China: A population-based cohort study | 2021 | It does not differentiate between ischemic or hemorrhagic stroke |
| A comprehensive analysis of cardiovascular mortality trends in Peru from 2017 to 2022: Insights from 183,386 deaths of the national death registry | 2023 | It does not differentiate between ischemic or hemorrhagic stroke |
| Cirugía de revascularización de miocardio usando arteria mamaria interna bilateral. Resultados a corto plazo | 2018 | It does not differentiate between ischemic or hemorrhagic stroke |
| The Prevalence and Correlates of Frailty in Urban and Rural Populations in Latin America, China, and India: A 10/66 Population-Based Survey | 2018 | It does not differentiate between ischemic or hemorrhagic stroke |
| Asociación entre submarinismo y trastornos neurocognitivos en personal militar retirado | 2018 | It does not differentiate between ischemic or hemorrhagic stroke |
| Contribution of chronic diseases to disability in elderly people in countries with low and middle incomes: a 10/66 Dementia Research Group population-based survey | 2009 | It does not differentiate between ischemic or hemorrhagic stroke |
| The contribution of chronic diseases to the prevalence of dependence among older people in Latin America, China and India: A 10/66 Dementia Research Group population-based survey | 2010 | It does not differentiate between ischemic or hemorrhagic stroke |
| Associations between DSM-IV mental disorders and subsequent non-fatal, self-reported stroke | 2015 | It does not differentiate between ischemic or hemorrhagic stroke |
| Time-series analysis of ambient PM(2.5) and cardiorespiratory emergency room visits in Lima, Peru during 2010-2016 | 2020 | It does not differentiate between ischemic or hemorrhagic stroke |
| Association between chronic conditions and health-related quality of life: differences by level of urbanization in Peru | 2017 | It does not differentiate between ischemic or hemorrhagic stroke |
| Sobredemanda de atención médica en el servicio de emergencia de adultos de un hospital terciario, Lima, Perú | 2016 | It does not differentiate between ischemic or hemorrhagic stroke |
| Identificación de síntomas relacionados al Síndrome de Apnea Obstructiva del sueño en historias clínicas de pacientes con eventos vasculares agudos | 2006 | It does not differentiate between ischemic or hemorrhagic stroke |
| Inhospital mortality in patients with type 2 diabetes mellitus: A prospective cohort study in lima, Peru | 2016 | It does not differentiate between ischemic or hemorrhagic stroke |
| Evolution of short cognitive test performance in stroke patients with vascular cognitive impairment and vascular dementia: Baseline evaluation and follow-up | 2017 | Duplicated |
| A Rare Case of Carotid Web in a Young Woman with Ischemic Stroke | 2021 | Duplicated |
| Covid-19 and Stroke in the Latin American Stroke Registry (LASE) | 2021 | Duplicated |
| on the epidemiology of cerebrovascular diseases in Peru | 1962 | Full-text not available |

**List of excluded references from updated search**

| **Title** | **Year** | **Reason to exclude** |
| --- | --- | --- |
| Metabolic status as a predictor of cardiovascular disease in a working cohort: beyond body mass index | 2025 | There is no information on stroke (neither population, nor variables, nor outcomes) |
| Left ventricle intracavitary thrombus in a patient with dilated cardiomyopathy | 2025 | There is no information on stroke (neither population, nor variables, nor outcomes) |
| Failure of Reconstructive Technique to Repair a Giant Intracranial Fusiform Aneurysm of the Basilar Artery: Case Report and Literature Review in the Pediatric Population | 2024 | Duplicated |
| Outcomes and Experiences of Patients and Their Caregivers After Severe Stroke Requiring Tube Feeding in Peru | 2024 | Duplicated |
| Aortic valve replacement through minithoracotomy. Results from the Peruvian experience | 2022 | Duplicated |
| Left sylvian subarachnoid neurocysticercosis: a therapeutic challenge | 2023 | Duplicated |
| Intra-arterial, aspirate and mechanical thrombectomy. First case in a patient with an acute stroke in a hospital | 2020 | Duplicated |
| Moyamoya disease: Chronic headache and progressive dementia associated with multiple brain strokes | 2019 | Duplicated |
| Takotsubo Syndrome: cardiovascular complication of stroke | 2022 | Duplicated |
| Acute cardiovascular complications in a Peruvian population of oncology patients | 2022 | Duplicated |
| Characteristics of interventions in cardiac surgery in a general hospital of Lima, Peru | 2022 | Duplicated |
| Trends in stroke mortality in Latin America and the Caribbean from 1997 to 2020 and predictions to 2035: An analysis of gender, and geographical disparities | 2025 | It does not differentiate between ischemic or hemorrhagic stroke |
| Global, regional, and national burden of disorders affecting the nervous system, 1990–2021: a systematic analysis for the Global Burden of Disease Study 2021 | 2024 | It does not differentiate between ischemic or hemorrhagic stroke |
| Concordance of diagnosis between statistical information and clinical history of patients with cardiopulmonary diseases | 2024 | It does not differentiate between ischemic or hemorrhagic stroke |
| ASSOCIATION BETWEEN LOW VITAMIN B12 LEVELS AND COGNITIVE IMPAIRMENT IN ELDERLY AT THE NAVAL MEDICAL CENTER OF PERU | 2023 | It does not differentiate between ischemic or hemorrhagic stroke |
| Perioperative morbidity and mortality of cardiac surgeries at the Instituto Nacional Cardiovascular, Lima, Peru, during the year 2023 | 2025 | It does not differentiate between ischemic or hemorrhagic stroke |
| Geospatial analysis of cardiovascular mortality before and during the COVID-19 pandemic in Peru: analysis of the national death registry to support emergency management in Peru | 2024 | It does not differentiate between ischemic or hemorrhagic stroke |
| Anthropometric Markers as Cardiovascular Predictors: A Comparison Between Conventional Cut-Off Points and Population Percentiles | 2025 | It does not differentiate between ischemic or hemorrhagic stroke |
| The burden of cardiovascular disease in Latin America and the Caribbean, 1990-2019: An analysis of the global burden of disease study | 2025 | It does not differentiate between ischemic or hemorrhagic stroke |
| Burden of disease attributable to high body mass index: an analysis of data from the Global Burden of Disease Study 2021 | 2024 | It does not differentiate between ischemic or hemorrhagic stroke |
| Burden of disease scenarios for 204 countries and territories, 2022–2050: a forecasting analysis for the Global Burden of Disease Study 2021 | 2024 | It does not differentiate between ischemic or hemorrhagic stroke |
| Survival and Mid-Term Outcomes of On-Pump vs. Off-Pump Coronary Artery Bypass Grafting: A Propensity Score-Matched Analysis in A First Peruvian Registry. | 2024 | It does not differentiate between ischemic or hemorrhagic stroke |
| Trends in Mortality Due to Stroke in South America between 1990 and 2019 | 2024 | It does not differentiate between ischemic or hemorrhagic stroke |
| Factors associated with adherence to antihypertensive agents in the older adult | 2024 | It does not differentiate between ischemic or hemorrhagic stroke |
| Economic burden of type-2 diabetes in Peru: a cost-of-illness study valuing cost differences associated with the level of glycemic control | 2024 | It does not differentiate between ischemic or hemorrhagic stroke |
| PROGNOSTIC CAPACITY OF D-DIMER IN PREDICTING MORTALITY IN PATIENTS DIAGNOSED WITH COVID-19 | 2024 | It does not differentiate between ischemic or hemorrhagic stroke |
| Global incidence, prevalence, years lived with disability (YLDs), disability-adjusted life-years (DALYs), and healthy life expectancy (HALE) for 371 diseases and injuries in 204 countries and territories and 811 subnational locations, 1990–2021: a systematic analysis for the Global Burden of Dise... | 2024 | It does not differentiate between ischemic or hemorrhagic stroke |
| Modified bentall and de bono surgery: Experience in a national hospital of Peru | 2020 | It does not differentiate between ischemic or hemorrhagic stroke |
| Neurocritical Care Organization in the Low-Income and Middle-Income Countries | 2025 | Includes population from Peru, but there are no individual results from them |
| Tobacco use and risk of acute stroke in 32 countries in the INTERSTROKE study: a case–control study | 2024 | Includes population from Peru, but there are no individual results from them |
| Global burden of 288 causes of death and life expectancy decomposition in 204 countries and territories and 811 subnational locations, 1990–2021: a systematic analysis for the Global Burden of Disease Study 2021 | 2024 | Includes population from Peru, but there are no individual results from them |
| Estimation of common carotid intima-media thickening using neural networks in adults with and without ischemic stroke | 2024 | Does not include population in Peru (Total from another country) |
| Stroke due to paradoxical cerebral embolism: case report of hereditary hemorrhagic telangiectasia | 2024 | Includes population from Peru, but there are no individual results from them |
| Availability and barriers to access post-stroke rehabilitation in Latin America | 2024 | Includes population from Peru, but there are no individual results from them |
| Stochastic Hybrid Algorithms to Estimate Stroke in Diabetic Patients | 2023 | Does not included humans |
| Self-Management to Anticipate Stroke in Diabetic Patients Through Algorithm Based on Probability of Bayes | 2023 | Does not included humans |
