# Supplementary material for: Ischemic stroke in Peru: a scoping review of trends, outcomes, and evidence gaps
Source: Front Epidemiol. 2026 Aug 28;6:1926861. doi: 10.3389/fepid.2026.1926861 (PMC13562258; doi:10.3389/fepid.2026.1926861)
Supplement: Supplementary file 2 [file Table2.docx]

**Figure S1. Pooled proportion** **of ischemic stroke among all stroke cases**

**Figure S2. Pooled proportion** **of women with ischemic stroke among all ischemic stroke cases**

**Figure S3. Pooled mean age of patients with ischemic stroke**

**Figure S4. Pooled proportion** **of ischemic stroke among other conditions**

**Figure S5. Pooled proportion** **of large artery atherosclerosis etiology among all ischemic stroke cases**

**Figure S6. Pooled proportion** **of cardioembolic etiology among all ischemic stroke cases**

**Figure S7. Pooled proportion** **of small vessel etiology among all ischemic stroke cases**

**Figure S8. Pooled proportion** **of other determined etiology among all ischemic stroke cases**

**Figure S9. Pooled proportion** **of undetermined etiology among all ischemic stroke cases**

**Figure S10. Pooled proportion** **of mortality among all ischemic stroke cases**

**Figure S11. Pooled proportion** **of tPA reperfusion rate among all ischemic stroke cases**
